# Evaluation of Choroidal Thickness in Type 2 Diabetes Using Spectral-Domain Optical Coherence Tomography

**DOI:** 10.22336/rjo.2025.28

**Published:** 2025

**Authors:** Shivapriya Manivannan, Avadhesh Oli

**Affiliations:** Department of Ophthalmology, Command Hospital (Air Force), Bangalore, India

**Keywords:** diabetes mellitus, diabetic retinopathy, macular edema, optical coherence tomography, choroidal thickness, DM = Diabetes Mellitus, DR = Diabetic Retinopathy, CT = choroidal thickness, SD-OCT = Spectral domain optical coherence tomography, NPDR = non-proliferative diabetic retinopathy, PDR = proliferative diabetic retinopathy, DME = diabetic macular edema

## Abstract

**Background and objectives:**

Microvascular changes induced by diabetes mellitus (DM) are the primary cause of diabetic retinopathy (DR) and choroidopathy. There is a lack of evidence linking diabetic retinopathy (DR) to changes in choroidal thickness (CT), so we designed this study to investigate this relationship. The choroidal thickness of DM patients, with or without DR, was compared to that of controls (subjects without diabetes) using spectral domain optical coherence tomography (SD-OCT).

**Materials and methods:**

We recruited 132 participants for a prospective observational study. Choroidal thickness at five points: subfoveal and at 500 and 1000 µm, both temporally and nasally to the fovea, was measured. OCT measurements, insulin use, lipid profiles, age, gender, fundus examination, and glycaemic control were recorded. The inferential and descriptive statistics were applied.

**Results:**

When compared to DM patients without DR, CT showed a trend toward lower values; however, only CT at 1000 μm temporal to the fovea (300.25 ± 65.37 μm in control group 1, 304.82 ± 76.71 μm in group 2, and 271.84 ± 65.07 μm in DR group 3) reached statistical significance (p = 0.05). Each diabetic subgroup did not differ in sub-foveal choroidal thickness (SFCT) (p = 0.586). Patients without DME (289.53 ± 63.86 μm) and those with DME (289.83 ± 100.99 μm) had comparable SFCTs (p = 0.992).

**Discussion:**

When comparing diabetic patients with and without diabetic retinopathy to healthy controls, there are differences in CT (increased, decreased, or no change). The atrophy and dropout of the choriocapillaris in eyes with diabetic retinopathy may be the cause of the decrease in CT in DR patients that our study revealed.

**Conclusion:**

DR patients showed a statistically significant decrease in CT at the 1000 μm temporal to fovea choroidal subregion compared to DM patients without DR. These results suggest that diabetes induces pathological changes in the choroid, resulting in retinopathy.

## Introduction

A thorough understanding of diabetes is crucial given the rise in diabetes mellitus cases worldwide and the disease’s tendency to increase morbidity and mortality significantly. Microvascular complication like diabetic retinopathy constitutes the leading cause of blindness in the working age population in developed countries [1] and pose a considerable economic burden on the healthcare systems [2-4].

Initially, DR was believed to be a disease primarily affecting the retinal microvasculature. However, further studies have reported a significant loss of choriocapillaris in diabetic subjects [5]. Thus, the concept of diabetic choroidopathy became apparent. The choroid plays a pivotal role in the metabolic exchange to the avascular fovea. Doppler flowmetry has also demonstrated decreased choroidal perfusion in early diabetic retinopathy (DR) [6]. Histochemical analysis has revealed changes in choriocapillaris, with the posterior pole being affected more significantly than the peripheral choroid [5]. OCT with enhanced depth imaging facilitates better visualization of the choroid and enables a precise measurement of choroidal thickness [7,8].

SD-OCT has become the most sought-after imaging tool in diabetic retinopathy and choroidopathy. It works on the principle of low-coherence interferometry. OCT offers high-resolution cross-sectional images of retinal tissue. This accuracy enables the analysis of various characteristic features of DR, facilitating the monitoring of disease progression and treatment outcomes.

Studies on the relationship between choroidal thickness (CT) and diabetes, as well as its variation with different stages of diabetic retinopathy, have yielded conflicting results. While some studies have shown an increase in thickness [9,10], others have reported a decrease in choroidal thickness in diabetic patients [1,1-15]. Given the paucity of definitive evidence on changes in choroidal thickness associated with diabetes and diabetic retinopathy, we conceptualized a prospective observational study to investigate changes in choroidal thickness in diabetic retinopathy.

## Materials and methods

This prospective observational study was conducted at the Command Hospital Air Force, Bangalore, following approval of the study protocol by the Institutional Ethics Committee. It was ensured that the study performed adhered to the tenets of the Declaration of Helsinki. Informed consent was obtained from every patient.

A total of 132 subjects were recruited for the study, divided into three groups: Group 1, the control group with no diabetes (44 subjects); Group 2, diabetic patients without diabetic retinopathy (44 patients); and Group 3, diabetic patients with diabetic retinopathy (44 patients). They were further classified into mild, moderate, or severe non-proliferative diabetic retinopathy (NPDR) or proliferative diabetic retinopathy (PDR), with or without diabetic macular edema (DME), according to the International Clinical Diabetic Retinopathy (ICDR) and Diabetic Macular Edema Severity scale.

The inclusion criteria for our study participants were as follows: 1) Volunteers above 18 years without diabetes presenting to the eye department, 2) Diabetic patients above 18 years of age without history of ocular treatment for diabetic retinopathy, such as retinal LASER or intraocular injection, like anti-VEGF/ steroids. Patients were excluded in the presence of any of the following conditions: 1) Hypertension, 2) Other vitreoretinal and choroidal disorders, 3) Patients on drugs likely to alter choroidal thickness, like immunosuppressive drugs, biologic therapies and potentially toxic drugs to the retina, choroid and/or optic nerve, 4) Active intra ocular inflammation/inflammatory diseases, 5) History of vitreoretinal surgery/cataract surgery in the past 6 months/complicated cataract surgery, 6) Spherical equivalent of refractive error ≥ -6 DS in myopes and ≥ +3 DS in hypermetropies, 7) Poor quality of OCT image resulting from significant media opacity like dense cataract or central corneal opacity showing poor quality OCT, 8) Patients with AMD, CSCR, PCV in fellow eye as these may affect choroidal thickness in the testing eye.

The demographic details of the patients were noted. Laboratory parameters, including fasting blood sugar (FBS), postprandial blood sugar (PPBS), HbA1c, lipid profile, and renal function tests, were sought. All subjects underwent a comprehensive ophthalmic examination that included visual acuity testing, intraocular pressure (IOP) measurement using a non-contact tonometer (Topcon CT-1P, Topcon, Tokyo, Japan), slit lamp examination to assess the anterior segment, and indirect ophthalmoscopy.

Digital fundus fluorescein angiography (FFA) was performed as needed.

Initially, the DM patients were divided into two groups based on the presence or absence of DR. If patients were found to have DR, they were further classified into mild, moderate, severe NPDR, or PDR with or without DME as per the ICDR and Diabetic Macular Edema Severity scale, and further subgroup analysis was also done.

**Fig. 1:**
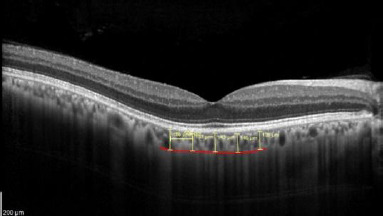
Objective quantification of choroidal thickness on EDI OCT image

All SD-OCT measurements were performed using the Heidelberg Spectralis (version 7.0.4, Heidelberg Engineering, Heidelberg, Germany). The value of central retinal thickness (CRT) was adopted from automated software quantification of the entire retina from the internal limiting membrane (ILM) to the posterior border of the Bruch membrane (**Fig. 1**).

**Fig. 2:**
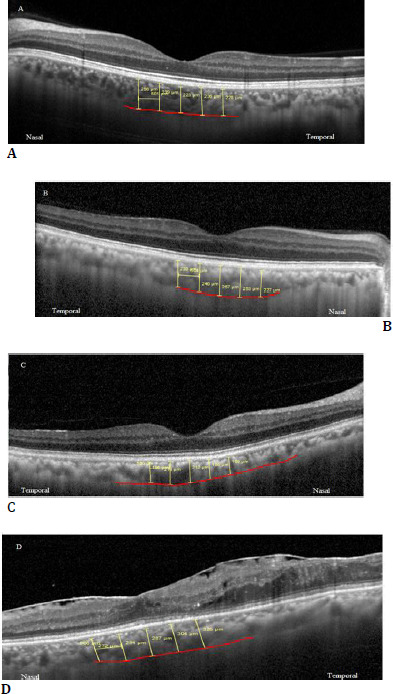
Representative OCT scan from (**A**) a normal subject; patient with (**B**) NPDR; (**C**) PDR; (**D**) DME

The choroid was imaged using the enhanced-depth imaging (EDI) mode centered at the fovea, which sets the choroid closer to the zero-delay line and thus theoretically provides better visualization of the choroidal-scleral interface. A high-precision software caliper was used to manually measure the choroidal thickness. It was measured perpendicularly from the outer edge of the retinal pigment epithelium to the choroidoscleral boundary at five points: temporal perifoveal (1000 μm temporal to the fovea), and temporal parafoveal (500 μm temporal to the fovea), sub foveal, nasal perifoveal (500 µm nasal to the fovea), nasal perifoveal (1000 µm nasal to the fovea) (**Fig. 2**).

Only data from one eye per subject was used for statistical analysis, which was randomly selected.

SPSS (Statistical Package for the Social Sciences) version 21 (IBM SPSS Statistics, IBM Corporation, NY, USA) was used for the statistical analysis. Appropriate descriptive and inferential statistics were used. P-values of ≤ 0.05 were accepted as being statistically significant.

## Results

**Table 1** shows the basic demographic and clinical features of the participants. Demographic analysis revealed that 63 (47.7%) patients were male and 69 (52.3%) were female, indicating nearly equal representation of males and females. Their average age was 58.02 ± 10.93 years, the mean weight was 67.28 ± 10.68 kg, and the average duration of diabetes was 10.56 ± 7.95 years. Of the 132 patients, 44 (33.33%) were without diabetes mellitus (DM) (Group 1), comprising 18 males (40.90%) and 26 females (59.1%); the average age was 56.8 ± 11.98 years. Their mean weight was 67.07 ± 10.82 kg. Of the 44 patients (33.33%) without diabetic retinopathy (DR) (Group 2), 18 (40.90%) were male and 26 (59.1%) were female. The average age was 54.64 ± 11.22 years, and the mean weight was 69.11 ± 10.86 kg. Of the 44 patients (33.33%) with DR (Group 3), 27 (61.40%) were male, 17 (38.6%) were female, and the average age was 62.61 ± 7.73 years. The mean weight of this group was 65.66 ± 10.30 kg. Participants with diabetes-related complications (DR) were more likely to be older than individuals in the other two groups (P < 0.05). However, no significant differences were found between the three groups in terms of gender and weight. Participants with DR had longer diabetes duration, higher FBS, PPBS, HbA1C, and BUN (all P < 0.05) compared to those without DR. In addition, a significantly higher number of patients with DR were on Insulin and had undergone cataract surgery (P < 0.05) when compared to other groups. The different parameters were similar among the control group, non-DR group, and DR group, including best-corrected visual acuity (BCVA), intraocular pressure, cholesterol, triglycerides, high-density lipoprotein (HDL), low-density lipoprotein (LDL), very-low-density lipoprotein (VLDL), and serum creatinine (all P > 0.05).

**Table 1:** Demographic and clinical features of the included participants

		Group 1	Group 2	Group 3	P Value
Characteristics	Overall	NO DM	No DR	Any DR	
**No. of subjects**	132	44 (33.33%)	44 (33.33%)	44 (33.33%)	
**Male**	63 (47.7%)	18 (40.90%)	18 (40.90%)	27 (61.40%)	0.085
**Female**	69 (52.3%)	26 (59.1%)	26 (59.1%)	17 (38.6%)	
**Mean age, year**	58.02 ± 10.93	56.8 ± 11.98	54.64 ± 11.22	62.61 ± 7.73	**0.002**
**Weight, kg**	67.28 ± 10.68	67.07 ± 10.82	69.11 ± 10.86	65.66 ± 10.30	0.314
**Duration of Diabetes, years**	10.56 ± 7.95	0	5.86 ± 4.689	15.30 ± 7.778	**0.001**
**Insulin**	43	0 (0%)	8 (18.2%%)	35 (79.5%%)	**0.001**
**OHA**	45	0 (0%)	36 (18.2%%)	9 (20.5%%)	
**Phakic**	112 (84.8%)	41 (93.2%)	41 (93.2%)	30 (68.2%)	**0.001**
**Pseudophakic**	20 (15.2%)	3 (6.8%)	3 (6.8%)	14 (31.8%)	
**BCVA**	0.23 ± 0.25	0.22 ± 0.27	0.18 ± 0.22	0.3 ± 0.26	0.063
**IOP**	17.45 ± 3.00	17.77 ± 2.7	17.68 ± 2.22	16.89 ± 3.85	0.317
**FBS**	136.94 ± 63.48	92.48 ± 8.87	151.94 ± 64.67	166.41 ± 69.81	**0.001**
**PPBS**	187.21 ± 91.39	116.79 ± 23.14	208.45 ± 84.95	236.39 ± 98.41	**0.001**
**HbA1C**	7.63 ± 2.29	5.48 ± 0.16	8.24 ± 2.06	9.18 ± 2.04	**0.001**
**Serum Cholesterol**	179.62 ± 41.42	182.16 ± 33.2	183.27 ± 37.43	173.43 ± 51.61	0.478
**HDL**	49.55 ± 12.89	52.35 ± 15.13	49.58 ± 10.36	46.73 ± 12.39	0.124
**LDL**	102.96 ± 34.09	108.31 ± 21.79	100.96 ± 35.77	99.61 ± 41.72	0.439
**VLDL**	33.05 ± 27.56	36.42 ± 38.37	30.05 ± 15.09	32.7 ± 24.36	0.556
**TG**	143.94 ± 81.59	132.77 ± 54.88	146.02 ± 68.49	153.04 ± 111.18	0.500
**Serum Creatinine**	1.24 ± 1.39	0.86 ± 0.29	1.32 ± 2.19	1.54 ± 0.89	0.064
**BUN**	143.94 ± 81.59	13.86 ± 8.32	13.05 ± 12.18	20.76 ± 9.92	**0.001**

Data are expressed as the mean ± standard deviation (SD) or as a percentage (%). Bold indicates statistical significance.

In terms of retinal thickness, the average central subfield retinal thickness was 232 ± 57.92 μm for all participants, 219.98 ± 22.14 μm for the control group, 217.23 ± 17.20 μm for patients without diabetic retinopathy (DR), and 258.80 ± 91.26 μm for patients with DR. Thus, the average RT of DR patients was significantly thicker than that of those without DR (258.80 ± 91.26 vs. 217.23 ± 17.20, P = 0.001) (**Table 2**).

**Table 2:** Comparison of the central sub-foveal CT of NPDR and PDR using an independent sample t-test

	Parameters	NPDR	PDR	Mean diff	p value
Choroidal thickness	1000 μm Temporal to fovea	274 ± 68.53	262.13 ± 48.93	11.87	0.646
	500 μm Temporal to fovea	282.28 ± 66.22	271.25 ± 40.37	11.02	0.655
	Central sub-foveal CT	290.56 ± 72.05	285.13 ± 53.46	5.43	0.842
	500 μm Nasal to fovea	279.19 ± 71.31	283.88 ± 56.85	-4.68	0.863
	1000 μm Nasal to fovea	268.22 ± 77.08	284 ± 57.08	-15.77	0.589

Data are expressed as the mean ± SD.

Among measurements done in five subregions, the CT showed a trend toward lower values in DR patients when compared to DM patients without DR but only CT at 1000 μm temporal to fovea (300.25 ± 65.37 μm in control group; 304.82 ± 76.71 μm in no DR group and 271.84 ± 65.07 μm in DR group) achieved statistical significance (P = 0.05). However, CT measured at the following locations: 500 μm temporal to fovea, sub fovea, 500 μm nasal to fovea, 1000 μm nasal to fovea, was found to be higher, though it did not achieve any statistical significance (P = 0.13, P = 0.42, P = 0.33, P = 0.37 respectively.

Statistical analysis of CT at various subregions of the choroid revealed reduced CT in the temporal and subfoveal subfields in the PDR group compared to the NPDR group. However, the results were not statistically significant.

Analysis of **Table 3** suggests that the severity of diabetic retinopathy (whether mild, moderate, or severe NPDR or PDR) did not correlate with changes in choroidal thickness (p-value ≥ 0.05).

**Table 3:** Comparison of the mean central sub-foveal CT, 1000 μ N, and 500 μ N among the groups (based on DR changes) using ANOVA

	Parameters	Mild NPDR (n=1)	Moderate NPDR (n=23)	Severe NPDR (n=12)	PDR (n=8)	P Value
Choroidal thickness	1000 μm Temporal to fovea	303	282.08 ± 70.01	256.08 ± 67.65	262.12 ± 48.93	0.656
	500 μm Temporal to fovea	322	288.96 ± 66.36	266.17 ± 68.02	271.25 ± 40.37	0.654
	Central sub-foveal CT	324	299.7 ± 73.9	270.25 ± 69.79	285.13 ± 53.46	0.586
	500 μm Nasal to fovea	320	282.91 ± 72.27	268.67 ± 73.69	283.88 ± 56.85	0.55
	1000 μm Nasal to fovea	297	272.65 ± 75.02	284 ± 57.08	257.33 ± 86.05	0.527

Data are expressed as the mean ± SD.

The CT values at different regions indicated a trend where patients with DME tended to have higher CT values in the areas temporal to the fovea and at 500 μm nasal to the fovea. However, none of these differences reached statistical significance. Moreover, in the subfoveal region, the CT was almost identical between the two groups. The CT measured 1000 μm nasal to fovea showed lower values in the DME-present group (258.33 ± 98.28 μm) compared to the DME-absent group (273.11 ± 70.32 μm).

## Discussion

This study compared choroidal thickness using spectral-domain optical coherence tomography (SD-OCT) between individuals with diabetes and those without, and analyzed the risk factors associated with diabetic retinopathy.

Among the CT measurements in five subregions, CT showed a trend toward lower values in DR patients when compared to DM patients without DR. Still, only CT at 1000 μm temporal to the fovea achieved statistical significance. Similar to our study, several researchers have reported a decrease in choroidal thickness in patients with diabetic retinopathy (DR) [16-19]. This could be due to atrophy and dropout of the choriocapillaris in eyes with diabetic retinopathy [4]. On the contrary, many studies have shown an increase in CT [20-26] in DR. Gołębiewska et al. [27] did not find any statistically significant differences in choroidal thickness in any subregion of the choroid. Thus, discrepancies can be observed in the CT (increased, decreased, or no change) of diabetic patients with and without diabetic retinopathy (DR) compared to healthy controls.

Statistical analysis of CT at various subregions of the choroid revealed reduced CT in the temporal and subfoveal subfields in the PDR group compared to the NPDR group. However, the results were not statistically significant. No significant correlation was found between the sub-foveal CT and different grades of DR in our study.

Various studies have shown conflicting results on the relationship between SFCT and grades of DR, in a survey by Ambiya V et al. [28]. The SFCT was found to be significantly lower in patients with proliferative DR compared to those with non-proliferative DR. In contrast, few studies have found that SFCT decreases with the progression of DR severity. This could be due to atrophy and dropout of the choriocapillaris in eyes with diabetic retinopathy. An increase in SFCT with the increasing severity of DR, which decreased with progression to PDR, was observed by Ram Kumar Jaiswal [29] and Wang et al. [30]. Microvascular abnormalities leading to retinal and choroidal ischemia, which in turn cause VEGF secretion, mediating fluid leakage in the interstitial space, might be the cause of increased CT in DR.

Although we have reached some insightful conclusions, this study has certain limitations. First, the CT was measured by hand; this could lead to measurement errors that automated quantification can prevent. It would have been possible to better understand choroidal pathology by dividing the choroidal layers into choriocapillary, Sattler’s, and Haller’s, and choroidal volume assessment layers.

## Conclusion

In this study, choroidal thickness showed a trend toward lower values in diabetic retinopathy patients compared to those with diabetes but without diabetic retinopathy, which was statistically significant at the choroidal subregion 1000 μm temporal to the fovea. There was no difference in choroidal thickness among the different grades of diabetic retinopathy (DR), and diabetic macular edema did not influence choroidal thickness. Our results indicate that diabetes mellitus leads to pathological changes in the choroid and can be used to understand disease pathogenesis. This is in line with previously reported histopathologic observations. Thus, our study proves that changes in the choroid can be evaluated non-invasively by using SD-OCT. Advances in treatment methods can explore newer modalities to prevent blindness in these cases, and it is prudent to identify patients with choroidopathy before their vision is affected.
